# The Effects of Individual Psychotherapy in BDNF Levels of Patients With Mental Disorders: A Systematic Review

**DOI:** 10.3389/fpsyt.2020.00445

**Published:** 2020-05-19

**Authors:** Felipe Cesar de Almeida Claudino, Leonardo Gonçalves, Felipe Barreto Schuch, Hugo Roberto Sampaio Martins, Neusa Sica da Rocha

**Affiliations:** ^1^ Center of Clinical Research and Center of Experiamental Research, Hospital de Clínicas de Porto Alegre (HCPA), Post-Graduation Program in Psychiatry and Behavioral Sciences, Federal University of Rio Grande do SUl (UFRGS), Porto Alegre, Brazil; ^2^ Department of Sports Methods and Techniques, Federal University of Santa Maria (UFSM), Santa Maria, Brazil; ^3^ Department of Internal Medicine, Federal University of Health Sciences of Porto Alegre (UFCSPA), Porto Alegre, Brazil

**Keywords:** psychotherapy, brain derived neurotrophic factor, BDNF, mental disorders, systematic review

## Abstract

**Background:**

Brain-derived Neurotrophic Factor (BDNF) is considered the main cerebral neurotrophin and is produced in the central neural system and peripherals. Its levels are reduced in patients with several psychiatric disorders, but it is unclear if the response to psychotherapy can alter its concentration.

**Objective:**

To carry out a systematic review evaluating the effects of individual psychotherapy in BDNF levels in patients with mental disorders.

**Methods:**

The databases PubMed, EMBASE, PsycArticles, SciELO, Web of Science, and CENTRAL; the last search was performed on October 2019 for trials evaluating the effects of individual psychotherapy in BDNF levels in adults with mental disorders. PROSPERO registration: CRD42018108144.

**Results:**

Eight of 293 studies were included. A rise in BDNF levels was observed in depressive patients when psychotherapy was combined with medication. Patients with post-traumatic stress disorder (PTSD) who responded to therapy presented a raise in BDNF levels mostly when combined with physical activity. There was a rise in BDNF levels in those who responded to psychotherapy in patients with bulimia, in borderline patients, and in insomniacs.

**Conclusions:**

The BDNF seems to present variations after psychotherapy especially in patients with bulimia, PTSD, insomnia, and borderline. These subjects also have symptom reduction. Thereby, BDNF could be a supplemental tool to analyze the success to psychotherapy. BDNF levels in patients with major depression after therapy are still controversial and the short follow-up of most studies is a limiting factor.

## Introduction

Psychotherapy is a well-grounded treatment for mental disorders with outcomes similar to pharmacotherapy (1). Psychotherapeutic interventions consist of exposure to stimuli, content resignification, and behavioral changes via interpersonal interaction (2). It acts on cognition and leads to symptoms remission, the formation of new neural networks, and can consequently lead to changes in demeanor (3). The neurotrophins are peptides in the central nervous system, and the most abundant is brain-derived neurotrophic factor (BDNF) (4), which stands out among the responsible factors in the formation of new neural networks that result in improved symptomatology.

BDNF is a neurotrophin that is more concentrated in certain regions of the brain such as the pre-frontal cortex and the hippocampus (5)—regions where complex cognitive processes occur including memory, personality, and emotional control. Therefore, psychotherapy stimulates these areas (6) reflecting on psychological symptom remission and resulting in a rise in BDNF levels.

Peripheral BDNF concentrations are lower in people with neuropsychiatric and neurodegenerative diseases (7, 8) versus matched controls. There is also signaling interference of this peptide in limbic areas related to emotion such as the hippocampus (9) that may contribute to the maintenance of the diseases.

BDNF levels also rise after antidepressant treatment, and increase in BDNF are associated with symptom improvements (10, 11); thus, it might be a potential mediator of the antidepressant treatment (12). Beyond pharmacological treatment, it is possible to observe increases in this neurotrophin's levels via electroconvulsive therapy (13) and physical activity (14) concurrent to the remission of psychological symptoms. Previous reviews of psychotherapies showed few potential response biomarkers for this treatment in patients diagnosed with PTSD such as the 5-alfa-reductase, heart rate changes (15), glucocorticoid metabolism, gene methylation, (16) and structural brain changes (17).

Despite the promising effects of psychotherapy response biomarkers, there is no systematic review evaluating the effects of individual psychotherapy in BDNF levels. Therefore, the objectives of this study are to: 1) evaluate if there are any changes in BDNF levels in people with mental disorders following individual psychotherapy and 2) evaluate if these changes are associated with symptom improvement.

## Methods

### Study Eligibility

This review adhered to the Preferred Reporting Items for Systematic Reviews and Meta-Analyses (PRISMA) (18). The protocol was registered on PROSPERO: CRD42018108144.

### Inclusion Criteria

The study population defined: patients over 18 years old, both genders, previously diagnosed with psychiatric disorders and/or mental disorders by qualified professionals and with its pathology described in the Diagnostic and Statistical Manual of Mental Disorders (DSM V).

The interventions of interest for the study: in-person individual psychotherapy: cognitive behavioral therapy, psychoanalysis, eye movement desensitization and reprocessing (EMDR), dialectical behavior therapy, interpersonal psychotherapy, analytically-oriented psychotherapy, and acceptance/commitment therapy, without limit of sessions or length of treatment.

Control groups defined: patients with no mental disorders or patients with psychiatric disorders submitted to other comparative treatments that are not psychotherapy, such as physical activity, meditation, and electroconvulsotherapy.

For the outcome, BDNF levels and disorder symptoms (according to symptom scales) were measured at two different times before and after psychotherapy exposure. Only serum or plasma BDNF levels were considered.

We included longitudinal randomized or non-randomized clinical trials as well as prospective and retrospective cohort studies; there was no language restriction, and any year of publication was considered including studies in progress.

### Exclusion Criteria

Patients undergoing only group therapy, online therapy, those without pre and post intervention BDNF serum or plasma levels, and patients who did not have their symptoms evaluated pre and post intervention were not included. Cross-sectional or case reports were not considered.

### Search Strategy and Study Selection

The titles and/or abstracts were obtained by two independent evaluators (FC and HM) by searching the following databases in October 2019: PubMed, EMBASE, PsycArticles, Scielo, CENTRAL, and Web of Science. The following terms were used for the research in PubMed: (“cognitive behavioral therapy” OR CBT OR “cognitive behavior therapy” OR “cognitive behavior treatment” OR “cognitive-behavioral treatment” OR “cognitive behavior therapy” OR “cognitive behaviour treatment” OR “cognitive behavioral therapy” OR “cognitive behavioural treatment”) OR [(psychotherapy OR “psychotherapeutic processes”) OR “Cognitive Therapy” OR (“psychotherapy, brief” OR “short-duration psychotherapy”) OR “interpersonal therapy” OR “analytical psychotherapy” OR “eye movement desensitization and reprocessing” OR EMDR] AND (“brain derived neurotrophic factor” OR BDNF OR neurotrophin OR neurogenesis OR “nerve cell plasticity” OR “brain plasticity” OR “nerve plasticity” OR “neural plasticity” OR neuroplasticity). The terms were adjusted according to the protocols in each database.The searches in each database were realized twice by two independent evaluators, adapting pre-defined terms following the protocol for each base (such as Mesh terms for PUBMED and thesaurus for Embase, for example). In any divergence regarding the abstracts of each database, an independent third party realized a new search. Independent search results were confronted and divergences were resolved by a third independent party. Abstracts from “grey literature” were only considered if available in the evaluated databases.

The study selection was conducted in two steps. First, the title and abstract of the articles obtained in the search were analyzed by two independent authors (FC and HM) and selected according to the inclusion and exclusion criteria. Next, the remaining articles were read in full. A third reviewer was recruited in case of any disagreement on the inclusion or exclusion of studies at any stage.

### Data Extraction and Analysis

Two authors, independently, extracted data on the following metrics, previously defined in pilot forms: year of publication, country, study design, patient precedence, sex, number of participants, disorder type, therapy type, therapy length, total of sessions, BDNF (plasma or serum), mean and standard deviation of BDNF levels (pre and post intervention), symptom measures, and complementary therapies (pharmacotherapy or else).

### Assessment of Risk of Bias

Two instruments were used to evaluate the “Assessment of risk of bias”: The New Castle-Ottawa (19) was indicated for longitudinal non-randomized studies. It evaluates aspects such as selection, comparability, and outcome (**Table 1**). Its scores vary between 0 (highest bias) to 9 points (lowest bias). The Cochrane Collaboration's tool assessed risk of bias for randomized studies (**Table 2**) (28). This allows evaluation as “high”, “low”, or “unclear” risk of bias for each domain: selection, attrition, detection, performance, reporting, and others. The intra-study risk of bias was also included (**Table 3**). There was no exclusion of articles, regardless of the score, due to the small number of studies obtained, but the restrictions were considered when analyzing the individual results.

**Table 1:** Evaluation of the methodological quality of longitudinal studies using the Newcastle-Ottawa scale.

Study	Selection				Comparability	Outcome			Total
	Representativeness	Selection of the non exposed	Ascertainment	Outcome of interest was not present at start of study	Comparability	Assessment of outcome	Follow-up long enough	Adequacy of follow up	
Koch et al. (20)	*		*		**	*		*	6
Yamada et al. (21)	*		*		**	*		*	6
Park et al. (22)	*		*		**	*		*	6
Perroud et al. (23)	*	*	*		**	*		*	7
Rusch et al. (24)	*		*		**	*	*	*	7

*Correspond to criteria (1 point).

**Correspond to criteria (2 points).

**Table 2:** Evaluation of the methodological quality of randomized studies using Cochrane Collaboration's tool for assessing risk of bias for studies.

	Silva et al. (25)	Powers et al. (26)	Yan et al. (27)
**Domain**			
**Random sequence generation**	Low risk	Low risk	Low risk
**Allocation concealment**	Low risk	Low risk	Low risk
**Blinding of participants and personnel**	Low risk	Low risk	Low risk
**Blinding of outcome assessment**	Low risk	Low risk	Low risk
**Incomplete outcome data**	Low risk	Low risk	Low risk
**Selective reporting**	Low risk	Low risk	Low risk
**Other sources of bias**	Low risk	Low risk	Low risk

**Table 3:** Evaluation of Intra-study bias risk of studies included.

Study	Clear definition of the study population?	Clear definition of results and evaluation?	Independent results evaluation? (by a third party, for example)	Sufficient follow-up time?	Selective loss during follow up?	Identified and clear limitations?
Koch et al. (20)	YES	YES	NO	NO	NO	YES
Silva et al. (25)	YES	YES	NO	YES	NO	YES
Powers et al. (26)	YES	YES	NO	YES	NO	NO
Yamada et al. (21)	YES	YES	NO	NO	NO	YES
Park et al. (22)	YES	YES	NO	NO	NO	YES
Perroud et al. (23)	YES	YES	NO	NO	NO	YES
Yan et al. (27)	YES	YES	NO	YES	NO	YES
Rusch et al. (24)	YES	YES	NO	YES	NO	YES

### Searches Results

**Figure 1:**
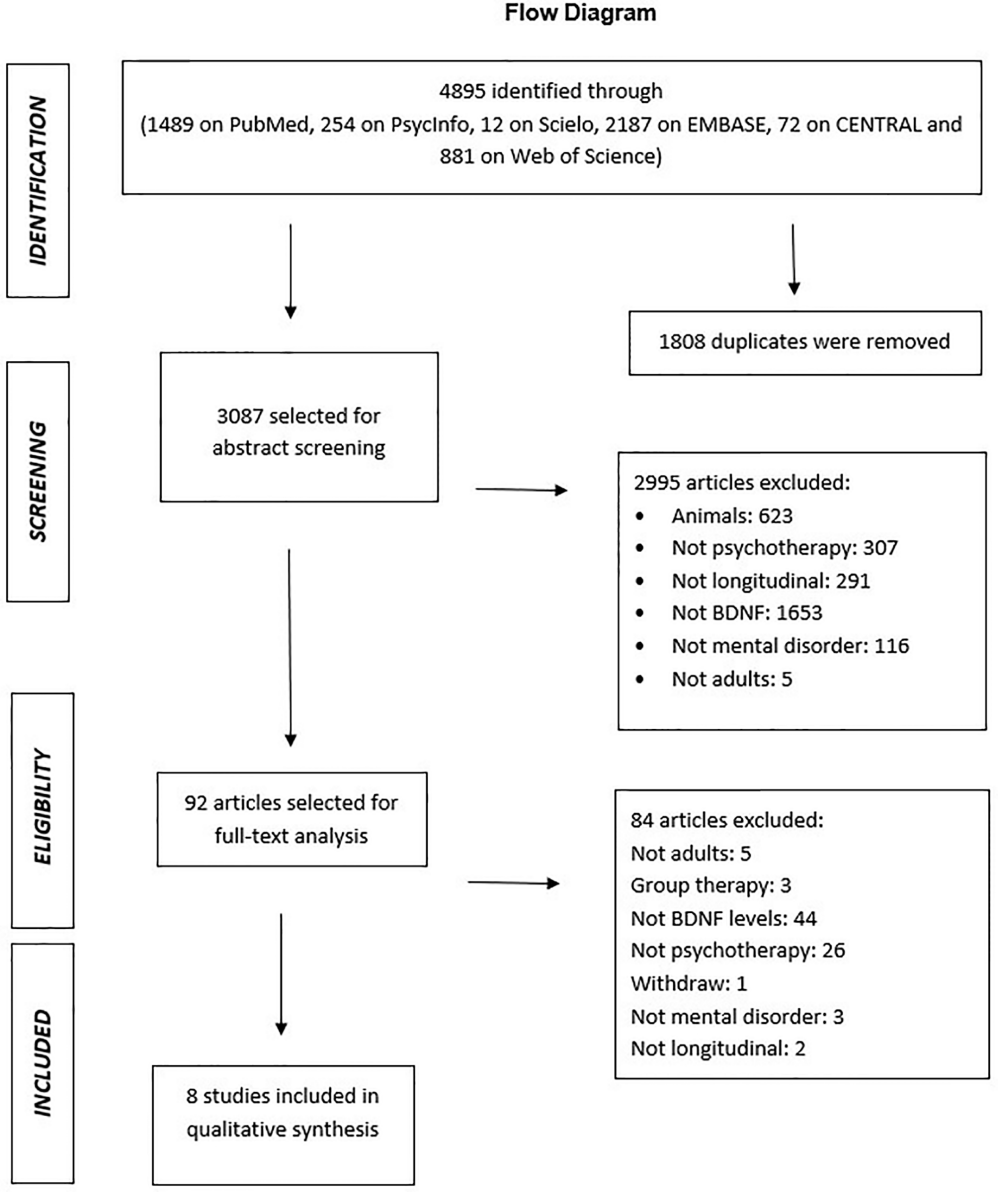
PRISMA flowchart of article selection (18).

A total of 4,895 references were found. Of these, 1,808 were duplicates and excluded. Thus, 3,087 abstracts were evaluated in terms of title and abstract followed by exclusion of 2,995 by criteria shown in the figure below. At the full-text stage, 92 articles were read in full, and 8 articles were added to the review and inspected followed by two proofreaders (**Figure 1**).

### Main Mental Disorders Studied

The main features of the studies are detailed in **Table 4**. We found studies evaluating BDNF levels in people with major depressive disorder (k=3), PTSD (k=2), bulimia (k=1), borderline disorder (k=1), and sleep disorder (K=1) with a predominance in women in five studies (55, 5–100%); three studies have a predominance of male patients. The number of patients varied between n=7 and n=115. Most patients came from outpatient clinics (k=4), and the treatment extent was 4 weeks to 12 months.

**Table 4:** Characteristics of included studies.

Study	N (M/F)	Design	Complementary Therapy	Setting patient	Time	BDNF extraction	Clinical Measure
Koch et al. (20)	27 (12/15)	Not randomized	Without medication	Inpatient and outpatient	12-16 sessions (6 weeks)	Plasma	HAMD
Silva et al. (25)	42 (7/35)	Randomized clinical trial	Without medication	Outpatient	16 sessions (12 weeks)	Serum	BDI-II
Powers et al. (26)	9 (1/8)	Randomized clinical trial	ET + Exercise psychopharmacological	No information	12 sessions (12 weeks)	Plasma	PSSI
Yamada et al. (21)	8 (8/0)	Not randomized	Without medication	Outpatient	4 weeks	Plasma	EDI, BDI and STAI
Park et al. (22)	7 (0/7)	Not randomized	Without medication (*)	No information	8 sessions	Plasma	CGI
Perroud et al. (23)	115 (7/108)	Not randomized	psychopharmacological	Outpatient	4 weeks	Plasma	BDI, BIS-10, CTQ, SCID-II
Yan et al. (27)	41 (22/19)	Randomized clinical trial	psychopharmacological	No information	12 months	Serum	HAMD
Rusch et al. (24)	44	Not randomized	APAP psychopharmacological	Outpatient	4-8 biweekly	Plasma	PSQI, QIDS-SR, PCL-M, SF-36

APAP, automatic positive airway pressure; BDI II, Beck Depression Inventory II; BDI, Beck Depression Inventory; BIS-10, Barrat Impulsiveness Scale; CGI-CS, Clinical Global Impression Change Scale; CTQ, Childhood Trauma Questionnaire; EDI, Eating Disorder Inventory; ET, Exposure therapy ; HAMD, Hamilton Depression Rating Scale; PCL-M, PTSD Checklist-Military Version; PSIQI, Pittsburgh Sleep Quality Index; PSSI, PTSD Symptom Scale-Interview; QIDS-SR, Quick Inventory of Depressive Symptomatology; SCID-II, Screening Interview for Axis II Disorder; SF-36, Short Form Health Survey-36; STAI, State–Trait Anxiety Scale. (*) 2 mg lorazepam or 10 mg diazepam was allowed for sleep management.

### Main Clinical Measures Evaluated and Diagnosis Criteria

The diagnostic criteria of the Diagnostic and Statistical Manual of Mental Disorders IV (29, 30) (k=3), SIDES (Structured Interviews for Disorders of Extreme Stress) (31) (k=1), Mini International Neuropsychiatric Interview (MINI-PLUS) (32) (k=1), Classification and Diagnostic Criteria for Mental Disorder in China 3 (CCMD-3) (33), and Epworth Sleepiness Scale (ESS) (34) were used. Different scales were used for the symptom evaluation: Hamilton Depression Rating Scale (35), (BDI) Beck Depression Inventory (36, 37), PTSD Symptom Scale-Interview Version (38), State–Trait Anxiety Scale (39), Clinical Global Impression Change Scale (CGI-CS) (40), (BIS-10): Barrat Impulsiveness Scale (41), (CTQ): Childhood Trauma Questionnaire (42), (SCID-II): Screening Interview for Axis II Disorder (43), (PSIQI): Pittsburgh Sleep Quality Index (44), (QIDS-SR): Quick Inventory of Depressive Symptomatology (45), (PCL-M): PTSD Checklist-Military Version (46), and (SF-36): Short From Health Survey-36 (47).

All studies presented symptom reduction on these scales. Three articles branched the patients into responders depending on the reduction of 50% of the HAMD (20) or BDI (23). baseline and CGI-C scores above the “much improved” level after therapy (22)

### Complementary Psychopharmacotherapy

Three of the articles included psychopharmacotherapy as a complementary therapy. Those diagnosed with insomnia could use automatic positive airway pressure (APAP). Only one study included physical activity versus complementary therapy (19). Six of the eight studies analyzed plasma levels of BDNF, and two opted for serum levels.

### Psychotherapy, Scales, and BNDF Levels

The main results of individual studies are described in **Table 5**. No significant changes in BDNF levels were observed in depressive patients and those who submitted to interpersonal therapy: [baseline responders (mean ±SD): 3.3±.3.7 x non-responders 2.8±1.3 p=0.68; Day 21-responders: 3.4 ±3.6 x non-responders 3.1±2.3 p=0.97] (17) or Cognitive-Behavioral Therapy [pre-treatment (mean ±SD) 1.387±0.26; post-treatment 1.328±0.3, p= 0.294] (18). However, the responders initially had a higher level of neurotrophin and a higher increase versus non-responders (17). In a study of medication associated with therapy, the group using only vortioxetine had a significant difference in BDNF levels between intervention and control groups in baseline and after. The group who underwent CBT associated with medication had a significant increase versus the control group (p<0.001) (27).

**Table 5:** Main results.

Study	Diagnosis	Therapy	MAIN RESULTS
(20)	Major Depressive Disorder	(IPT)	17 patients had a reduction of the least 50% on the baseline Hamilton scale score and were defined as “responders” after psychotherapy. BDNF had no meaningful difference between the responders and non-responders groups. Age, sex, HAMD, subject as inpatient or outpatient, number of previous depressive events, or pharmacological treatment before the therapy had no meaningful correlation with BDNF levels. There were no association between BDNF levels and depression severity
(25)	Major Depressive Disorder	(CBT)	There was a significantly depression symptoms reduction after psychotherapy. There were no significant differences between pre and post psychotherapy intervention BDNF levels.
(26)	Post-traumatic stress disorder	(ET)	Psychotherapy, as only treatment, in patients with PTSD did not change BDNF levels in 12 weeks. Exposure therapy associated with physical activity increased the BDNF levels in patients.
(22)	Post-traumatic stress disorder	(EMDR)	There were no meaningful changes on BDNF plasma levels after psychotherapy, but responders presented higher BNDF plasma levels than non-responders. Anxiety, phobia, and dissociation levels were significantly reduced after EMDR. BDNF basal levels presented correlation with the depression and anxiety estimated response
(21)	Bulimia Nervosa	(CBT)	BDNF levels in patients with bulimia increased after treatment. There were no differences between BDNF levels in inpatients and outpatients There was a reduction in the frequency of self-induced vomiting, laxatives using, and compulsive eating episodes after therapy. There were no significant changes on the Beck Depression Inventory and Eating Disorder Inventory scores, except for “Drive for Thinness”.
(23)	Borderline Disorder	(I-DBT)	Plasma BDNF levels in subjects with BPD were higher than in the control group There was an inversely proportional decrease of BDNF levels in response to psychotherapy Non-responders had a reduction of BDNF levels after psychotherapy and, responders, had an increase, both, not significant.
(27)	Major Depressive Disorder	(CBT)	After the treatment, the group that underwent pharmacotherapy combined with psychotherapy presented a most expressive depressive symptom reduction in comparison to the submitted only to the pharmacological treatment. Before the treatment, both control and intervention group had BDNF levels with no significant differences. After intervention, the group with psychotherapy associated presented a significantly higher increase of BDNF
(24)	Sleep Disorder	(CBT)	The group that had an improvement on sleep patterns had a not significant BDNF increase, while in the group that its sleep patterns got worse, BDNF levels did not change.

CBT, Cognitive-Behavioral Therapy; EMDR, Eye Movement Desensitization and Reprocessing; ET, Exposure Therapy; I-DBT, Intensive dialectical behavior therapy; IPT, Interpersonal therapy.

Two studies (19, 28) in PTSD patients have shown increased levels of BDNF after intervention in different modalities: exposure therapy (19) and eye movement desensitization and reprocessing (28). The first study related elevation of neurotrophin when psychotherapy was associated with physical activity (mean ±SD) (pre: 1.38 x post: 3.73). Their values were not expressive in isolation (pre: 1.77 x post: 1.75) and PSSI: PE (prolonged exposure therapy): (mean ±SD) 37.00 (8.25); PE+ Exercise: 42.00 (5.2) (19). However, the second study indicated that patients who presented a clinical response or symptom remission symptoms had higher increases in BDNF than those who did not respond to treatment [responders: (mean ±SD 4435.6±1273.4; non-responders 2789±643.2; p 0.025)]. There was an association between the increase of BDNF levels and symptoms reduction such as anxiety, phobia, and dissociation after EMDR in patients with PTSD (19).

At baseline, patients with bulimia nervosa undergoing eye movement desensitization and reprocessing had reduced BDNF values versus controls (p = 0.02)—this has been established in previous studies. This sample presented elevation of BDNF levels which were more expressive among responders than non-responders. The pre and post therapy scale scores of responders are listed here: BDI pre (mean ±SD) 22±11.9, post: 17.4±15.2, p=0.22, STAI-state pre: 56±12.9, post 50±17.7, p=0.07, STAI-trait pre: 65.8±10.1, post: 55.2±18.5, p 0.08 (13).

Borderline personality diagnosed patients had a higher BDI mean than the healthy control group (mean ± SD 34.10±11.8) similar to other symptom scales. Responders had an insignificant increase in plasma BDNF levels (p=0.062), as non-responders had insignificant reduction (p=0.78) (23).

Finally, patients with sleep disorders were divided between those who presented an improved sleep pattern and those who got worse. The first group presented an insignificant BDNF increase (pre: 80.2± 28.6; post 89.1±36.3; p =0.089). BDNF levels were stable in the group with no improvement (pre: 91.7±38.1; post 100.2±44.4; p=0.155) (27).

## Discussion

To the best of our knowledge, this is the first systematic review to evaluate the relation between BDNF levels in response to psychotherapies. The results showed that, in general, there was a reduction of clinical symptoms in patients with mental disorders that went through different kinds of psychotherapies. In most cases, there was also a concurrent rise in BDNF levels.

In central nervous system, the levels of BDNF are higher in structures of the limbic system, such as the hippocampus (48) and, in patients with mental disorders, the neurotrophin concentration in this region is reduced (49). Meanwhile, the psychotherapy has the potential for stimulation of the limbic system and (50), although the physiology is not clear until this moment, we can assume que psychotherapies act in this system, stimulating a higher BDNF production, reducing psychiatric symptoms.

While this present review showed that there is no absolute consensus regarding BDNF levels rise after psychotherapy, there is meta-analysis that shows evidence of the increase of BDNF after pharmacological treatment (51, 52). Although both treatments are recognized as effective, this difference can be explained by the short follow-up time of the patients. The response to pharmacological therapy tends to occur faster (53), while the response to psychotherapy may take months (54), depending on factors such as therapeutic relation, for example (55).

These findings showed initial evidence that BDNF could be a potential tool to evaluate the effect of psychotherapies on patients with mental disorders. The BDNF levels could be used with symptom evaluation and other tools such as clinical symptom scales to confirm that there is a satisfactory response to therapy. Furthermore, BDNF levels tend to be stable over time (56), and psychotherapy can be a variable course. Thus, such levels might change at a different rate versus those observed here. Only one of our manuscripts had a follow-up longer than 12 weeks. Prior reviews and meta-analyses have shown that there is an increase in BDNF levels in depressive patients treated with pharmacologically (57) or with physical activity (58). Healthy patients have stable BDNF levels over time, which favors the theory that patients with mental disorders submitted to therapies have an increase in neurotrophins as an outcome. However, in this review, only patients using medication associated with psychotherapy had an increased BDNF.

Although there is initial evidence for the role of BDNF as an individual psychotherapy response biomarker, the heterogeneity across the studies limit the conclusions of this meta-analysis—the groups had different disorders, types of therapies, therapy exposure times, and others boundary conditions that may affect the results. The literature is restricted to depression, PTSD, bulimia, insomnia, and borderline disorders. Thus, the conclusions cannot be extended to other disorders like bipolar disorder and anxiety disorders.

We also cannot disregard the relevance of polymorphism knowledge in response to psychotherapies. A biological response marker could also be expanded to other biomarkers. Longitudinal studies evaluating BDNF levels in response to psychotherapy could facilitate the performance of a meta-analysis of such candidate biomarkers.

### Limitations

The conclusions of this review must be cautious because the studies included in this review are small, most of which with a short follow up period. As to BDNF values, the included studies quantified serum and plasma BDNF levels and there is evidence that the levels of these molecules change according to analyzed tissue and organs (59, 60) Also, it isn't possible to quantify the proBDNF precursor and its derivatives values: mature BDNF and pro-peptide BDNF. It is relevant to know this data, for there is evidence that they have different effects in the physiopathology of psychiatric disorder (61). As well, age (62) and ethnicity (63) may interfere in BDNF levels and also were not considered while quantifying the neurotrophin.

## Conclusion

In conclusion, there is a nascent body of evidence evaluating the effects of individual psychotherapies on BDNF. These neurotrophins seem to present variations after psychotherapy especially in patients with bulimia, PTSD, insomnia, and borderline personality, and that show reductions in symptoms. In patients with depression, those who submitted only to psychotherapy had no increase in BDNF levels while patients with associated medicine usage showed an obviously higher increase versus those who submitted solely to pharmacological treatment. There is only one study with a higher than 12-week follow-up period, which suggests that a longer follow-up time is needed for BDNF levels. BDNF could supplement symptom scales to analyze the effects of psychotherapy.

## Data Availability Statement

The data set obtained for this study are available from corresponding author on request.

## Author Contributions

FC: preparation and registration of the review protocol, extraction of articles in the databases, selection of articles, analysis of results, and article production. LG: protocol production, results analysis, and article production. FS: protocol production, results analysis, and article production. HM: preparation of the review protocol, extraction of articles in the databases, selection of articles, analysis of results, and article production. NR: main orientation, protocol production, review of articles in case of divergence, analysis of results, and article production.

## Funding 

This study was financed in part by the Coordenação de Aperfeiçoamento de Pessoal de Nível Superior–Brasil (CAPES)—Finance Code 001 and by Hospital de Clínicas de Porto Alegre Research Incentive Fund (FIPE).

## Conflict of Interest

The authors declare that the research was conducted in the absence of any commercial or financial relationships that could be construed as a potential conflict of interest.
